# Medium & long-chain acylcarnitine’s relation to lipid metabolism as potential predictors for diabetic cardiomyopathy: a metabolomic study

**DOI:** 10.1186/s12944-021-01576-9

**Published:** 2021-11-02

**Authors:** Dan-meng Zheng, Zhen-ni An, Ming-hao Ge, Dong-zhuo Wei, Ding-wen Jiang, Xue-jiao Xing, Xiao-lei Shen, Chang Liu

**Affiliations:** 1 Department of Endocrinology, The First Affiliated Hospital of Jinzhou Medical University, No. 2, section 5, Renmin Street Guta District, Jinzhou, Liaoning Province China; 2 Department of Orthopedic, The First Affiliated Hospital of Jinzhou Medical University, No. 2, section 5, Renmin Street Guta District, Jinzhou, Liaoning Province China; 3 Department of Endocrinology, Liaoning University of Traditional Chinese Medicine, No.79, Chongshan East Road, Huanggu District, Shenyang, Liaoning Province China

**Keywords:** Diabetic cardiomyopathy, Metabolomics, Acylcarnitine, Lipid metabolism, AMPK

## Abstract

**Background:**

Acylcarnitine is an intermediate product of fatty acid oxidation. It is reported to be closely associated with the occurrence of diabetic cardiomyopathy (DCM). However, the mechanism of acylcarnitine affecting myocardial disorders is yet to be explored. This current research explores the different chain lengths of acylcarnitines as biomarkers for the early diagnosis of DCM and the mechanism of acylcarnitines for the development of DCM in-vitro*.*

**Methods:**

In a retrospective non-interventional study, 50 simple type 2 diabetes mellitus patients and 50 DCM patients were recruited. Plasma samples from both groups were analyzed by high throughput metabolomics and cluster heat map using mass spectrometry. Principal component analysis was used to compare the changes occurring in the studied 25 acylcarnitines. Multivariable binary logistic regression was used to analyze the odds ratio of each group for factors and the 95% confidence interval in DCM. Myristoylcarnitine (C14) exogenous intervention was given to H9c2 cells to verify the expression of lipid metabolism-related protein, inflammation-related protein expression, apoptosis-related protein expression, and cardiomyocyte hypertrophy and fibrosis-related protein expression.

**Results:**

Factor 1 (C14, lauroylcarnitine, tetradecanoyldiacylcarnitine, 3-hydroxyl-tetradecanoylcarnitine, arachidic carnitine, octadecanoylcarnitine, 3-hydroxypalmitoleylcarnitine) and factor 4 (octanoylcarnitine, hexanoylcarnitine, decanoylcarnitine) were positively correlated with the risk of DCM. Exogenous C14 supplementation to cardiomyocytes led to increased lipid deposition in cardiomyocytes along with the obstacles in adenosine 5′-monophosphate (AMP)-activated protein kinase (AMPK) signaling pathways and affecting fatty acid oxidation. This further caused myocardial lipotoxicity, ultimately leading to cardiomyocyte hypertrophy, fibrotic remodeling, and increased apoptosis. However, this effect was mitigated by the AMPK agonist acadesine.

**Conclusions:**

The increased plasma levels in medium and long-chain acylcarnitine extracted from factors 1 and 4 are closely related to the risk of DCM, indicating that these factors can be an important tool for DCM risk assessment. C14 supplementation associated lipid accumulation by inhibiting the AMPK/ACC/CPT1 signaling pathway, aggravated myocardial lipotoxicity, increased apoptosis apart from cardiomyocyte hypertrophy and fibrosis were alleviated by the acadesine.

## Introduction

The incidence of diabetes is increasing year by year [1]. However, with the ever-growing number of diabetes, the macro-complications of diabetes such as diabetic cardiomyopathy (DCM) are also increasing. DCM remains the leading cause of death associated with diabetes [2, 3]. DCM is an abnormality of specific myocardial structure with loss of function in diabetic patients, independent of cardiomyopathy or other cardiovascular diseases such as coronary artery disease, hypertension, and so on [4, 5]. At present, the pathogenesis of DCM has not been fully clarified. However, the disease progression of DCM has mainly been divided into three stages: 1) the early stage of cell and metabolic dysfunction, with no apparent effect on systolic dysfunction; 2) the middle stage, where cell apoptosis initiates with a slight increase in left ventricular size, diastolic dysfunction, and left ventricular ejection fraction < 50%; 3) the late-stage, which is generally characterized by the systolic and diastolic dysfunction, microvascular injury, cardiovascular autonomic neuropathy, and eventually heart failure [6].

Energy metabolism inactivation in DCM patients is manifested as lipotoxicity and decreased metabolic flexibility associated with the early development of type 2 diabetes mellitus (T2DM) [7, 8]. The accumulation of cellular lipids and free fatty acids causes organic cardiac dysfunction in the middle and late stages as well as in the early phase of DCM, which is an early event of diabetic heart function deterioration [9, 10]. Cardiac lipotoxicity is caused by the damage of fatty acid β oxidative, particularly mitochondria overload disorders [11, 12]. Fatty acids are catalyzed by the endoplasmic co-enzyme network and the acyl-CoA synthase at the outer mitochondrial membrane to form acyl-CoA before being transported for oxidation and energy supply to the mitochondria through carnitine [13]. Abnormal fatty acid oxidation can be assessed by acylcarnitines, which are the intermediate oxidative metabolites consisting of esterified fatty acid of carnitine molecule.

Recently, the metabolic characteristics of amino acids and acylcarnitines are linked with the occurrence and the development of T2DM [14]. However, the relationship between acylcarnitine and DCM is still uncertain. Although a reliable biomarker has not yet been determined, acylcarnitine can be used as a potential biomarker in DCM development. However, the acylcarnitine effects on myocardial disorders during the development of DCM are not sufficiently clear. Therefore, the present study was carried out, based on mass spectrometry targeted and high-throughput metabolomics analysis on the plasma samples of T2DM, to explore the metabolic changes of acylcarnitine for its potentials as a biomarker for early diagnosis of DCM. In addition, this study also examined the acylcarnitine and its differential metabolites mechanism of action in the development of DCM on myocardial cells, further studying the correlation of acylcarnitine with myocardial lipid metabolism disorders myocardial hypertrophy, and fibrosis.

## Materials and Methos

### Patient data and research methods

#### Patient data

This prospective study included patients attending the First Affiliated Hospital of Jinzhou Medical University from January 2017 to January 2021. The Clinical Research Ethics Committee approved this study of the First Affiliated Hospital of Jinzhou Medical University. The participants were divided into simple type 2 diabetes mellitus (ST2DM) and DCM groups. Eligibility criteria for patients with DCM in this study were: (1) Age ≥ 18 years; (2) diagnosis of T2DM; (3) more than 5 years duration of T2DM; (3) cardiac expansion with contraction or diastolic dysfunction as detected by cardiac color Doppler ultrasound; (4) heart failure; and (5) early to late diastolic trans mitral flow velocity < 1. Exclusion criteria of the study were: (1) diagnosed as type 1 diabetes; (2) pregnancy; (3) heart failure due to coronary heart disease, hypertension, myocarditis, rheumatic heart disease, and/or other types of cardiomyopathies; and (4) acute infection, malignant tumor & cognitive impairment. Meantime, the clinical data including demographic index such as age, gender, body mass index (BMI), fasting blood glucose (FPG), systolic blood pressure (SBP), diastolic blood pressure (DBP), triglycerides (TG), total cholesterol (TC), low-density lipoprotein cholesterol (LDL-C), high-density lipoprotein cholesterol (HDL-C), glycated hemoglobin (HbA1c), postprandial blood glucose (PBG), and acylcarnitine metabolites of the enrollees were retrieved from the hospital’s electronic database.

Acylcarnitine metabolites were detected by metabolomics. The detailed detection methods are described in the previous studies [15]. Under fasting conditions, blood from the fingertip was collected from all subjects and placed onto the special filter paper plate and after that, evenly distributed dried blood spots were subjected for metabolomics analysis after the detection by the tandem mass spectrometer (AB Sciex, Framingham, MA, USA).

### Cell culture

H9c2 cells, which are rat cardiomyoblast cell lines, were obtained from the Newgainbio company. The cells were grown at 37 °C, and 5% CO2 in the atmosphere using Dulbecco’s modification of Eagle’s medium Dulbecco (DMEM) (4.5 g/L, D-Glucose, Gibco, Grand Island, NY, USA) supplemented with 10% fetal bovine serum, penicillin (100 units/ml), and streptomycin (100 ugs/ml) (Gibco, Grand Island, NY, USA). For establishing a model of myocardial insulin resistance and to simulate the pathogenesis of DCM in-vivo*,* the H9c2 cells were exposed to palmitic acid (PA) and DMEM [16]. The cells were grouped as the control, the PA, the C14, the C14 + Oxfenicine, and the C14+ acadesine (AICAR) group. *Control group*: no treatments were performed; *PA group*: incubation of H9c2 cells with 0.3 mM PA and high glucose medium for 24 h; *C14 group*: co-incubation of H9c2 cells with 25 μM C14 and high glucose medium for 24 h; *C14 + Oxfenicine group*: co-incubation of H9c2 cells with 25 μM C14 and 3 mM Oxfenicine with high glucose medium for 24 h [17]; *C14 + AICAR group*: co-incubation of H9c2 cells with 25 μM C14 and 1 mM AICAR with high glucose medium for 24 h [18].

### Preparation of PA

Fatty acid-free bovine serum albumin (BSA) and three-distilled water prepared 40% BSA mother liquor. Then PA was prepared in 0.01 mol/L NaOH solution to prepare a 40 mM PA storage solution in a water bath at 75 °C for 30 min. The 40 mM PA solution was quickly added to the 40% BSA solution, and after that, the solution was solubilized at 55 °C for 30 min. The dissolved solution was filtered at the workbench to obtain a 20 mM PA stock solution. For the experiments, the working concentration of PA was diluted with DMEM containing 10% fetal bovine serum. In contrast, the NaOH mixed with 40%BSA at the same concentration was used as a negative control.

### Cell viability assay

3-(4,5-Dimethylthiazol-2-yl)-2,5-diphenyltetrazolium bromide (MTT) assay was used to evaluate the cytotoxicity of H9c2 incubated with PA and C14. At 37 °C and 5% CO_2_ in the atmosphere, the H9c2 were incubated with gradient concentration of either PA (0.01, 0.03, 0.05, 0.2, 0.3, 0.5, 0.6, 0.8, 1.0 mM) or C14 (5, 10, 20, 25, 30, 50, 70, 85,100 μM) for 24 h. Following the incubation, 20 μl MTT solution (5 mg/mL) was added to cells for 4 h. Afterward, the supernatant was discarded, and 150 μl dimethyl sulfoxide was added to cells for 10 min. A microplate reader was taken to record the absorbance at 490 nm.

### Measurement of glucose intake

According to the manufacturer’s instructions, glucose uptake was measured using 2-NBDG (N13195, Thermo Fisher Scientific, USA). 0.3 mM PA was added to cells for 24 h, following the treatment with 50 μm 2-NBDG in a glucose-free medium for 30 min. Finally, the microplate reader was used to record the fluorescence intensity absorbance.

### Western blotting

Western blotting analysis was performed [19], and bands intensity was calculated using ImageJ software. Anti-Bcl-2 (AF6139), anti-pan-AKT1/2/3 (AF6261), anti-phospho-pan-AKT1/2/3(Ser473) (AF0016), anti-IL-1β (AF5103), anti-Cleaved-Caspase3 (AF7022), anti-TNF-α (AF7014), anti-Bax (AF0120), anti-MMP9 (AF5228), anti-CPT1b (AF3094) antibodies were purchased from Affinity biosciences in Jiangsu, China. Whereas anti-Collagen I (ab260043) antibody was purchased from Abcam in Shanghai, China. Anti-AMPKα (2532S), anti-TGFβ (3711S), anti-ACC (3662S), anti-p-ACC (Ser79) (3661S), and anti-p-AMPKα (Thr172) (2535S) were purchased from Cell Signaling Technology in Shanghai, China. Anti-MYHC (sc-376,157) was purchased from Santa Cruz in Shanghai, China. Finally, an imaging system (Tanon, Shanghai, China) was used to detect and visualize the signal.

### Immunofluorescent dual-labeling staining

After H9c2 cells were treated as described in 2.2, permeabilized with 0.1% Triton-X at room temperature for 15 min, and finally washed thrice with phosphate-buffered saline (PBS). The cells were incubated with goat serum for two hours at room temperature and then overnight in primary antibodies at 4 °C. The primary antibodies used are as follows: anti-CPT1b (1:500, Affinity Biosciences, AF3094); anti-TNF-α (1:500, Affinity Biosciences, AF7014); anti-Cleaved-Caspase3 (1:500, Affinity biosciences, AF7022,); anti-Bcl-2 (1:500, Affinity Biosciences, AF6139); anti-Collagen I (1:200, Abcam, ab260043); anti-MYHC (1:200, Santa Cruz, sc-376,157). The following day, the cells were then incubated with the secondary antibodies. The secondary antibodies used are as follows: goat anti-rabbit IgG or goat anti-mouse IgG (1:250, Alexa Fluor 488/594, Thermo Fisher Science, A- 11034/A-11005). The cells were additionally stained with 4′6-Diamidino-2-phenylindole (DAPI) for 15 min. Finally, the immune-stained cells were observed with a fluorescence microscope, and the fluorescence intensity was analyzed with ImageJ software.

### Oil red O staining analysis

The Cell Oil Red O (ORO) Staining Kit was purchased from Solarbio Biotechnology (Beijing, China). Briefly, the cells were incubated with ORO for 20–30 min. Then 60% isopropanol was added, and the cells were soaked for 5 min. After discarding 60% isopropanol, freshly prepared ORO was added to the cells for 10-20 min for the disseminated staining. Afterward, the staining solution was discarded. Then Mayer hematoxylin staining solution was added to the cells for 1-2 min to stain the nucleus. After the incubation, the staining solution was discarded, and cells were washed with water 2–5 times. Afterward, ORO Buffer was added for 1 min, and then the staining solution was discarded. Finally, distilled water was added to cover the cells and observed under an optical microscope for imaging. Cells were re-stained with iso-pharanol, and each well was measured with a microplate reader at 490 nm for quantifying the lipid deposition levels. The optical density represents the relative degree of lipolysis at 490 nm wavelength.

### Measurement of free fatty acid and cholesterol

In the H9c2 cells, the free fatty acids and triglycerides were measured with a commercial kit purchased by Solarbio Biotechnology (Beijing, China). The manufacturer’s instructions and calculation formulas were used to calculate free fatty acids and cholesterol levels, as provided by the commercial assay kits.

### Lipid peroxidation test

Amount of lipid peroxidation was detected by BODIPY™ 581/591 C11 (C10445; Thermo Fisher). The cells were directly stimulated with BODIPY™ 581/591 C11 for 30 min, and then the fluorescence was detected by confocal microscopy. This study used a method to perform Lipid peroxidation assay as described earlier [19].

### Detection of mitochondrial membrane potential and apoptosis and TUNEL assay

Mitochondrial Membrane Potential and Apoptosis Detection Kit One-step TUNEL cell apoptosis detection kit was used (from the Beyotime Institute of Biotechnology, Shanghai, China). The fluorescence intensity analysis was done by ImageJ software.

### Data analysis

Statistical Product and Service Solutions (SPSS) 26.0 software was used to process all the data. The Chi-square test or Fisher’s exact test was used to compare Categorical data between two groups. At the same time, the P-P plot tested the continuous variables for the normality tests. Continuous variables were first tested for normal distribution. If the data fit a normal distribution, they were presented as Means ± Standard deviation (SD) and compared using student’s t-test. However, If the data did not follow the normal distribution, it was presented with a median with interquartile range (IQR) and compared using the Wilcoxon signed-rank test. More than two groups of data were analyzed using one-way ANOVA. The data was also tested for homogeneity of variance. If the variances were equal, the Bonferroni post hoc test was performed. But in the case where the variance of the data was not equal, Mann- Whitney U-test was used for data analysis. Finally, factor analysis was used to compare the acylcarnitines. Multivariable binary logistic regression was applied to analyze odds ratios (OR) and 95% confidence intervals (CI) of the acylcarnitine factors extracted from groups. The value of *P* < 0.05 was considered statistically significant.

## Results

### Clinical data of patients

**Fig. 1:**
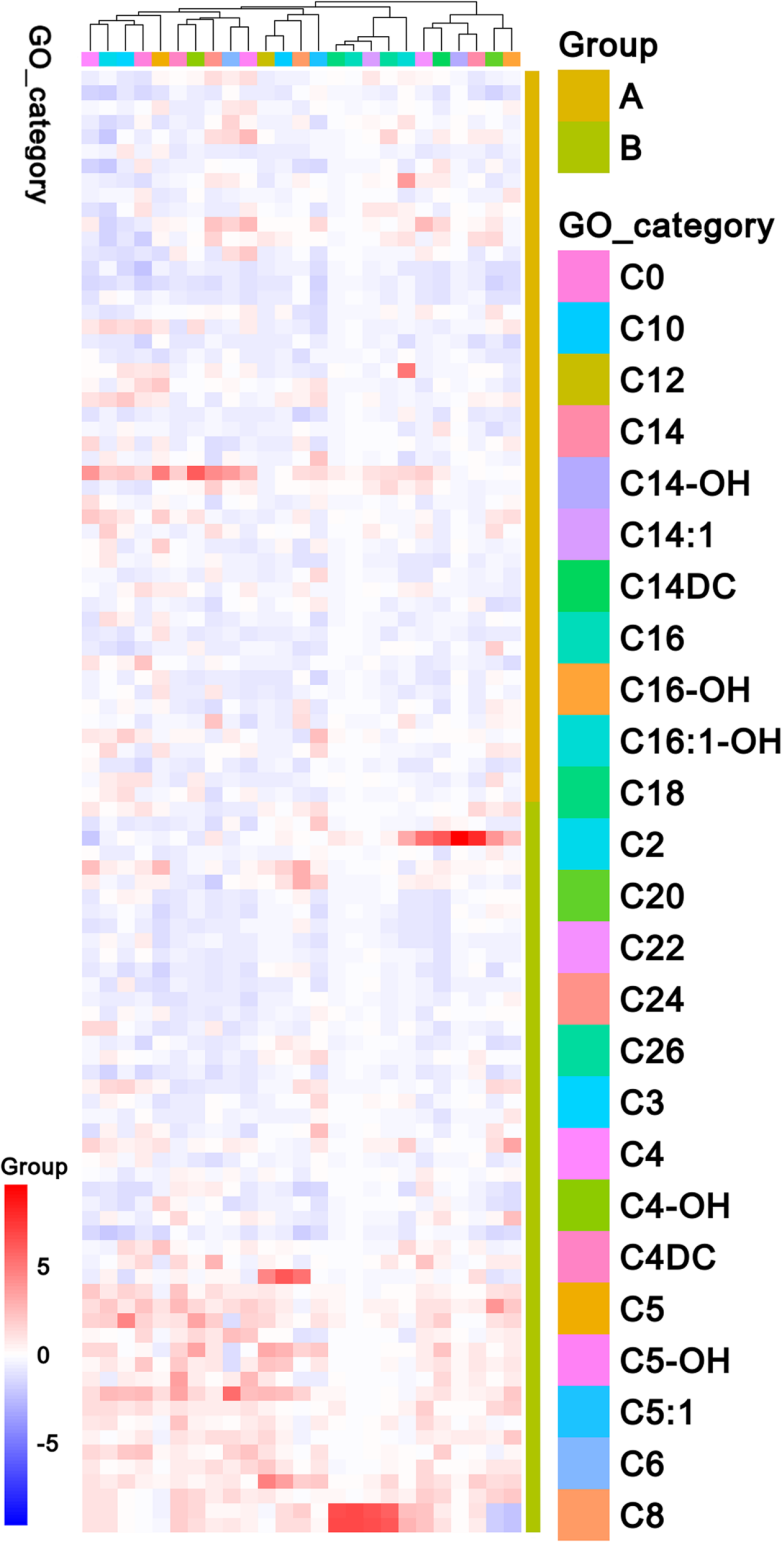
The heat map showing the changes in 25 acylcarnitines and their differences between the two groups. Group A represents the ST2DM group, and group B represents the DCM group. The heat map shows the expression of medium and long-chain acylcarnitine in group B (primarily red) and in group A (primarily blue)

This study included 100 participants, divided into the DCM (50 patients) and the ST2DM group (50 patients). The demographic description of the patients is shown in Table 1. Compared with the patients in the ST2DM, the patients in the DCM group were older and had higher FPG, PBG, HbA1c, and TG. However, no apparent discrepancies in BMI, SBP, DBP, TC, HDL-C, and LDL-C were observed between both groups. In the DCM group, C0, C2, C8, C10, C12, C14, C14-OH, C14DC, C14:1, C16, C16:1-0H, C20, C24, and C26 were higher than in the ST2DM group, while C5 levels were lower. However, other acylcarnitines were comparable in the two groups (Table 2). The heat map in Fig. 1 represents the trends and the detected difference observed for all metabolites in the two groups.

**Table 1:** Baselines characteristic of subjects

***Variables***	***DCM***	***ST2DM***	***P***
*N*	50 (50%)	50 (50%)	
Age, years	47.42 ± 7.43	44.08 ± 5.32	0.01*
Male, gender	22 (44%)	29 (58%)	0.161**
BMI, kg/m^2^	25.61 ± 3.44	24.71 ± 1.20	0.082*
SBP, mmHg	129 ± 6.92	127 ± 5.7	0.065*
DBP, mmHg	80 ± 12.60	79 ± 6.41	0.066*
FPG, mmol/L	8.96 ± 0.67	8.01 ± 0.47	< 0.001*
PBG, mmol/L	17.43 ± 1.42	16.60 ± 0.50	< 0.001*
HbA1c, %	8.75 ± 0.36	7.60 ± 0.39	< 0.001*
TG, mmol/L	1.73 ± 0.65	1.16 ± 0.38	< 0.001*
TC, mmol/L	4.34 ± 0.62	4.16 + 0.57	0.129*
LDL-C, mmol/L	2.40 ± 0.37	2.34 ± 0.42	0.452*
HDL-C, mmol/L	0.99 ± 0.12	1.02 ± 0.84	0.166*

All data are represented as n (%), means ± standard deviation

*p-values represent differences among groups as compared by Student’s t-test. **p-values represent differences among groups as compared by Chi-squared test

**Table 2:** Acylcarnitine profile in DCM patients

***Variables***	***DCM***	***ST2DM***	***P***
	***Median (interquartile)***	***Median (interquartile)***	
C0, μM	30.95 (24.77–36.84)	24.59 (20.45–31.54)	0.002
C2, μM	12.93 (10.43–16.40)	10.76 (8.87–15.36)	0.039
C3, μM	1.87 (1.43–2.30)	1.65 (1.13–2.18)	0.119
C4, μM	0.23 (0.16–0.34)	0.19 (0.15–0.24)	0.058
C4-OH, μM	0.05 (0.03–0.10)	0.05 (0.03–0.07)	0.275
C4DC, μM	0.41 (0.21–0.85)	0.30 (0.24–0.41)	0.088
C5, μM	0.13 (0.09–0.16)	0.14 (0.11–0.19)	0.042
C5-OH, μM	0.19 (0.13–0.28)	0.18 (0.14–0.26)	0.923
C5:1, μM	0.03 (0.02–0.07)	0.04 (0.02–0.06)	0.774
C6, μM	0.07 (0.06–0.09)	0.07 (0.05–0.08)	0.236
C8, μM	0.11 (0.07–0.17)	0.06 (0.04–0.08)	< 0.001
C10, μM	0.16 (0.08–0.27)	0.05 (0.03–0.07)	< 0.001
C12, μM	0.08 (0.06–0.10)	0.05 (0.03–0.07)	< 0.001
C14, μM	0.08 (0.05–0.11)	0.06 (0.04–0.08)	0.002
C14-OH, μM	0.04 (0.02–0.07)	0.03 (0.02–0.04)	0.016
C14DC, μM	0.03 (0.02–0.05)	0.02 (0.02–0.04)	0.362
C16, μM	1.02 (0.76–1.42)	0.78 (0.62–0.92)	< 0.001
C16-OH, μM	0.02 (0.02–0.03)	0.02 (0.02–0.03)	0.668
C16:1-OH, μM	0.05 (0.03–0.07)	0.04 (0.03–0.05)	0.030
C18, μM	0.54 (0.38–0.71)	0.48 (0.36–0.59)	0.133
C20, μM	0.03 (0.02–0.05)	0.02 (0.02–0.03)	0.012
C22, μM	0.06 (0.03–0.08)	0.05 (0.04–0.07)	0.319
C24, μM	0.04 (0.03–0.06)	0.03 (0.02–0.04)	0.036
C26, μM	0.03 (0.02–0.04)	0.03 (0.02–0.04)	< 0.001
C14:1, μM	0.10 (0.06–0.16)	0.06 (0.03–0.08)	< 0.001

The content of 25 acylcarnitines data, presented with a median with IQR and compared between groups using the Wilcoxon signed-rank test

### Extracted factors of Acylcarnitines

**Fig. 2:**
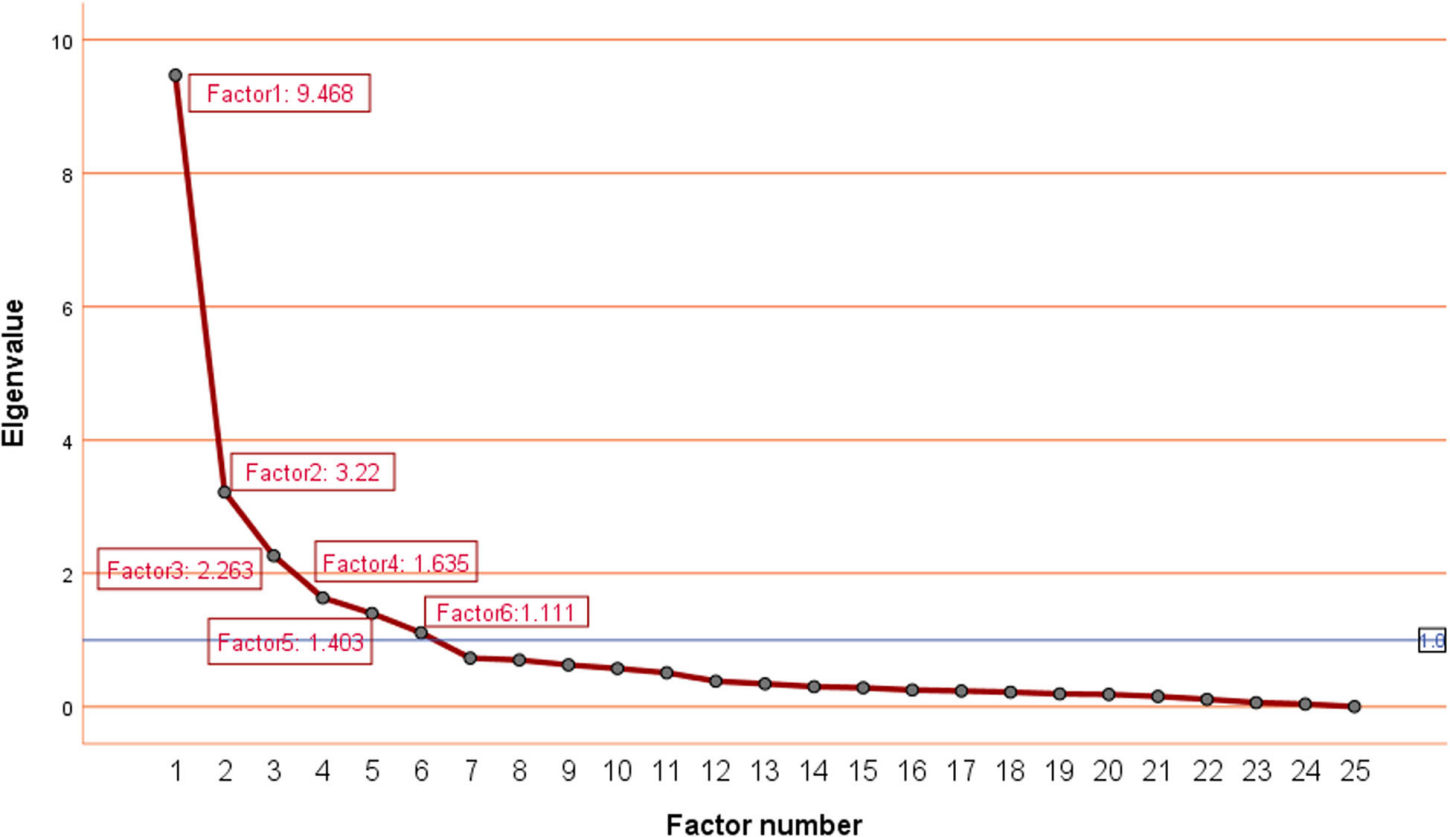
A Scree plot represents the eigenvalue and number of factors. The number of acylcarnitine factors was determined by the eigenvalue > 1 and the elements on the Scree plot. Factor 1 comprised C14, C12, C14DC, C14-OH, C20, C18, C16:1-OH; factor 2 comprised C26, C14:1, C24, C22, C4-OH; factor 3 comprised C2, C3, C4, C16; factor4 comprised C8, C6, C10; factor 5 included C5-OH, C4DC, C0; and factor 6 comprised C5, C16-OH

The high Kaiser-Meyer-Olkin coefficient of 0.898 and the *P*-value of Bartlett’s sphericity test < 0.0001 were significant for the extracted factors, confirming the acceptability of the factor analysis results is acceptable. Eigenvalue, community, and gravel plots were used to decide the number of acylcarnitine factors. The specific requirements for making the distinctions were as follows: eigenvalue > 1, community ≥50%, and factor number on the steep slope of gravel map. The eigenvalues of factors 1–5 were more significant than 1 and showed steep slope of the pebble map (Fig. 2). Therefore, this research extracted six factors and listed the acylcarnitine loadings on the five factors after varimax rotation, as listed in Table 3. Factor 1 comprised C14, C12, C14DC, C14-OH, C20, C18, C16:1-OH; factor 2 comprised C26, C14:1, C24, C22, C4-OH; factor 3 comprised C2, C3, C4, C16; factor4 comprised C8, C6, C10; factor 5 included C5-OH, C4DC, C0; and factor 6 comprised C5, C16-OH. All the studied six factors were explained 76.40% of the total variance.

**Table 3:** Factor and their loadings derived by 25 acylcarnitine metabolites

***Variables***	***Factor 1***	***Factor 2***	***Factor 3***	***Factor 4***	***Factor 5***	***Factor 6***
C14	**0.945**	−0.098	−0.070	0.066	0.019	0.002
C12	**0.881**	0.036	0.029	0.335	0.003	−0.010
C14DC	**0.735**	0.357	0.114	−0.109	0–.051	0.229
C14-OH	**0.729**	0.374	0.028	0.207	0.223	0.011
C20	**0.687**	0.515	0.047	0.123	0.192	0.047
C18	**0.624**	0.271	0.416	−0.046	0.137	−0.183
C16:1-OH	**0.603**	0.372	0.389	−0.067	0.276	0.159
C26	0.251	**0.739**	0.235	0.420	0.065	−0.156
C14:1	0.268	**0.735**	0.227	0.422	0.049	−0.133
C24	0.324	**0.706**	0.065	0.107	0.141	0.094
C22	0.219	**0.696**	0.070	−0.003	0.251	0.284
C4-OH	0.030	**0.667**	0.485	0.038	0.209	0.167
C2	0.040	0.105	**0.837**	0.180	0.121	−0.023
C3	0.122	0.025	**0.777**	0.058	0.250	0.134
C4	−0.093	0.349	**0.741**	0.169	0.159	0.222
C16	0.462	0.289	**0.605**	0.062	0.146	−0.264
C8	0.126	0.215	0.091	**0.905**	0.130	−0.044
C6	0.027	−0.031	0.136	**0.842**	−0.077	0.277
C10	0.147	0.357	0.169	**0.782**	0.240	−0.229
C5-OH	0.105	0.215	0.156	0.013	**0.849**	0.076
C4DC	0.086	0.331	0.213	0.073	**0.747**	−0.177
C0	0.082	−0.110	0.452	0.170	**0.681**	0.051
C5:1	0.049	0.466	0.082	0.076	0.476	0.306
C5	−0.022	0.125	0.493	0.094	0.109	**0.645**
C16-OH	0.490	0.211	−0.045	− 0.045	−0.055	**0.529**

Principal component analysis of extracted factors from 25 acylcarnitines according to eigenvalue > 1, lithotripsy, and cumulative variance. To maximize the variance difference of each factor, varimax rotation is used. Each black labeled acylcarnitine in each column defines a factor in the corresponding column (*factor 1*: C14, C12, C14DC, C14-OH, C20, C18, C16:1-OH; *factor 2*: C26, C14:1, C24, C22, C4-OH; *factor 3*: C2, C3, C4, C16; *factor4*: C8, C6, C10; *factor 5*: C5-OH, C4DC, C0; and *factor 6*: C5, C16-OH)

### Associations between extracted factors and DCM risk in T2DM

In the univariate analysis, factors 1, 2, 3, and 4 were positively correlated with DCM, however, factor 6 was negatively correlated with DCM. Model 2 demonstrates the analysis and adjustment of all aspects, but only factors 1 and 4 showed a significant positive relationship with DCM, with factor 6 negatively correlated with DCM. Other parameters such as sex, age, BMI, SBP, DBP, TC, HDL-C, LDL-C, PBG, and HbA1c were further adjusted in Model 3. The present study found that only factors 1 and 4 were significantly positively correlated to DCM, while factor 6 was negatively related to DCM (Table 4).

**Table 4:** Univariable and multivariable association of factors with DCM

***Model***	***Factor***	***OR***	***95%CI***	***P***
Model 1	Factor 1	5.73	2.53–12.96	< 0.001
	Factor 2	1.36	1.01–1.82	0.042
	Factor 3	1.42	1.06–1.90	0.018
	Factor 4	4.43	2.32–8.46	< 0.001
	Factor 5	0.98	0.74–1.29	0.877
	Factor 6	0.28	0.17–0.45	< 0.001
Model 2	Factor 1	7.08	1.73–28.96	0.007
	Factor 2	1.65	0.80–3.4	0.178
	Factor 3	1.32	0.81–2.16	0.268
	Factor 4	10.59	4.36–25.71	< 0.001
	Factor 5	0.89	0.58–1.36	0.581
	Factor 6	0.11	0.53–0.24	< 0.001
Model 3	Factor 1	10.09	2.78–36.58	0.01
	Factor 2	1.42	0.96–2.11	0.08
	Factor 3	1.47	0.99–2.18	0.056
	Factor 4	3.33	1.45–7.67	0.004
	Factor 5	0.73	0.48–1.12	0.152
	Factor 6	0.23	0.12–0.47	< 0.001

Model 1: Univariable model; Model 2: Multivariable model, adjusted for other

Acylcarnitine factors; Model 3: Multivariable model, further adjusted for sex,

body mass index, systolic blood pressure, diastolic blood pressure, total cholesterol, low density lipoprotein cholesterol, high density lipoprotein cholesterol, age, and postprandial blood glucose

### Tandem mass spectrometry results for the medium and Long-chain Acylcarnitine levels

**Fig. 3:**
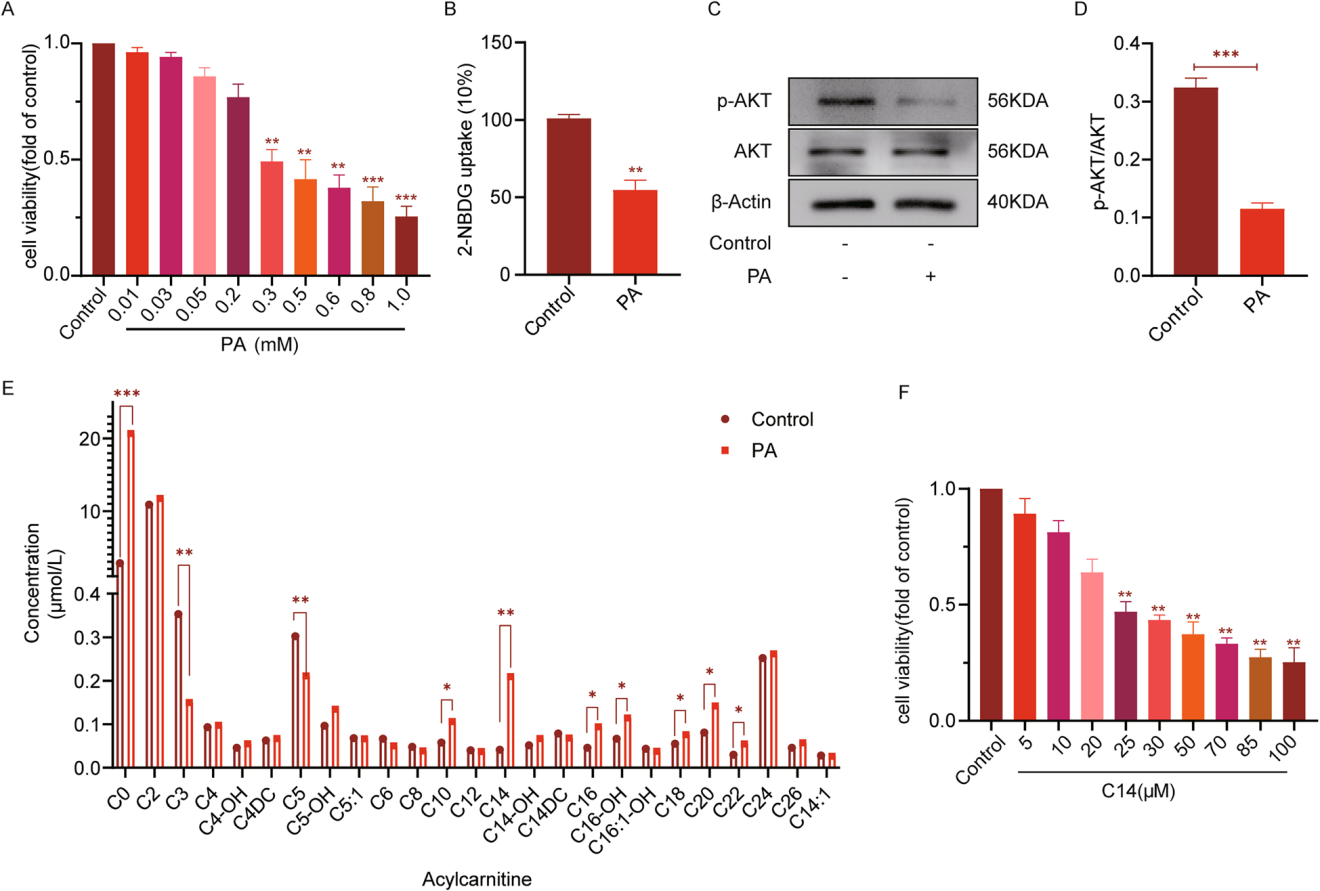
The results of tandem mass spectrometry showing the increased levels of medium and long-chain acylcarnitine. **A** MTT method was used to detect the survival rate of H9c2 after PA (0.01, 0.03, 0.05, 0.2, 0.3, 0.5, 0.6, 0.8, 1.0 mM) treatment for 24 h to verify the virulence of PA (*n* = 6). **B** Measurement of glucose uptake using 2-NBDG (*n* = 6). **C** The expressions of p-AKT, AKT in each group (n = 6). **D** The analysis results of p-Akt and Akt expression (*n* = 6). **E** Tandem mass spectrometry analysis was used to analyze the cells (*n* = 7, data is presented with a median and compared using the Wilcoxon signed-rank test between groups). **F** The survival rate was analyzed using MTT assay for H9c2 after C14 (5,10,20,25,30,50,70,85,100 μM) treatment for 24 h, to verify the virulence of C14 (*n* = 6). The rest of the data is represented as means ± SD, where two-way ANOVA followed by Tukey’s post hoc test was used. *P < 0.05, **P < 0.01, and ***P < 0.001

The results of MTT analysis showed that following 24 h, the survival rate of H9c2 cells was significantly inhibited, with the 50% reduction in the cell survival ability, with the treatment of PA at a concentration more than 300 μM. Therefore, 300 μM PA was selected to intervene in H9c2 cells to establish the insulin resistance model for the subsequent experiments (Fig. 3A). As shown in Fig. 3B, with respect to the control group, the glucose uptake in the PA group was markedly decreased. In line, p-AKT/AKT protein level in the PA group was also reduced, as observed through western blotting analysis (Fig. 3C-D), suggesting the successful establishment of the cellular insulin resistance model. The tandem mass spectrometry analysis of the cells showed that C0, C10, C14, C16, C16-OH, C18, C20, and C22 were significantly higher in the PA group compared with the control group. Among all of them, the C14 levels increased the most, however, decreases in the C3 and C5 levels were observed (Fig. 3E).

### C14 causes accumulation of lipids in cardiomyocytes with induction pro-inflammatory reactions to increase cell apoptosis

**Fig. 4:**
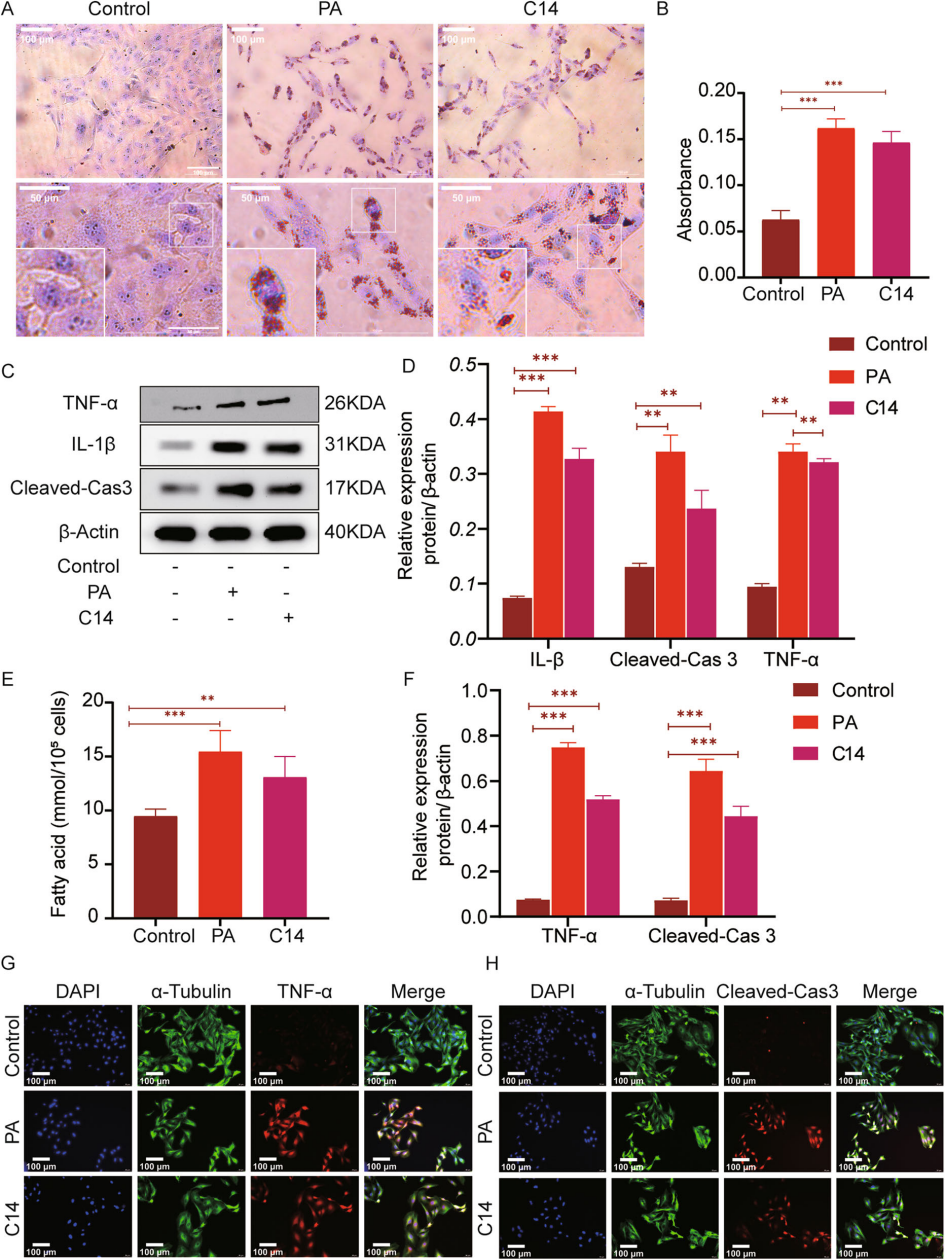
C14 caused the accumulation of lipids in cardiomyocytes and induced the pro-inflammatory mediators to increase the cell apoptosis. **A** ORO staining was used to observe the lipid deposition (*n* = 6, Scale bar = 50 μm, 100 μm). **B** Quantitative analysis of intracellular lipid droplets. **C** The expressions of TNFα, IL-1, and Cleaved-caspase3 in control, PA, and C14 group (*n* = 6), as detected using western blotting. **D** The results of TNFα, IL-1, and Cleaved-caspase3 expression. **E** The free fatty acids of H9c2 cells in each group was measured (n = 6) and compared with the treated and control group. **F** The data revealed positive expression of TNFα and cleaved-caspase 3 in H9c2. (G-H) Immunofluorescence staining results showing the levels of TNF-α and Cleaved-caspase3 in H9c2 (n = 6, Scale bar = 100 μm). Data are represented as means ± SD, where two-way ANOVA followed by Tukey’s post hoc test employed. *P < 0.05, **P < 0.01, and ***P < 0.001

The MTT analysis showed that after 24 h, the survival rate of H9c2 cells was significantly inhibited, with the 50% reduction in the cell survival ability, with the treatment of C14 at a concentration of more than 25 μM. Therefore 25 μM C14 was selected to intervene in H9c2 cells for the subsequent experiments (Fig. 3F). With respect to the control group, the intracellular lipid deposition in the PA and the C14 group increased variably, but here again, the PA group showed increased lipid deposition than the C14 group as analyzed through ORO staining (Fig. 4A-B). Further, compared to the control group, the levels of TNFα, IL-1, and Cleaved-caspase3 in the PA and C14 groups increased significantly (Fig. 4C-D). The free fatty acids of H9c2 cells in each group were measured, and the results showed that the NEFA level of the PA group and the C14 group increased variably. However, the PA group had a significant increase than the C14 group (Fig. 4E). Immunofluorescence analysis revealed that TNFα and Cleaved-caspase3 expression was much higher in the PA and C14 group comparing with the control group (Fig. 4G-H). Comparing with the control group, the number of positive cells in the PA and C14 groups were increased significantly. However, the number of positive staining cells in the PA group was higher than in the C14 group (Fig. 4F).

### C14 inhibit the AMPK/ACC/CPT1-b signaling pathway and lipid accumulation

**Fig. 5:**
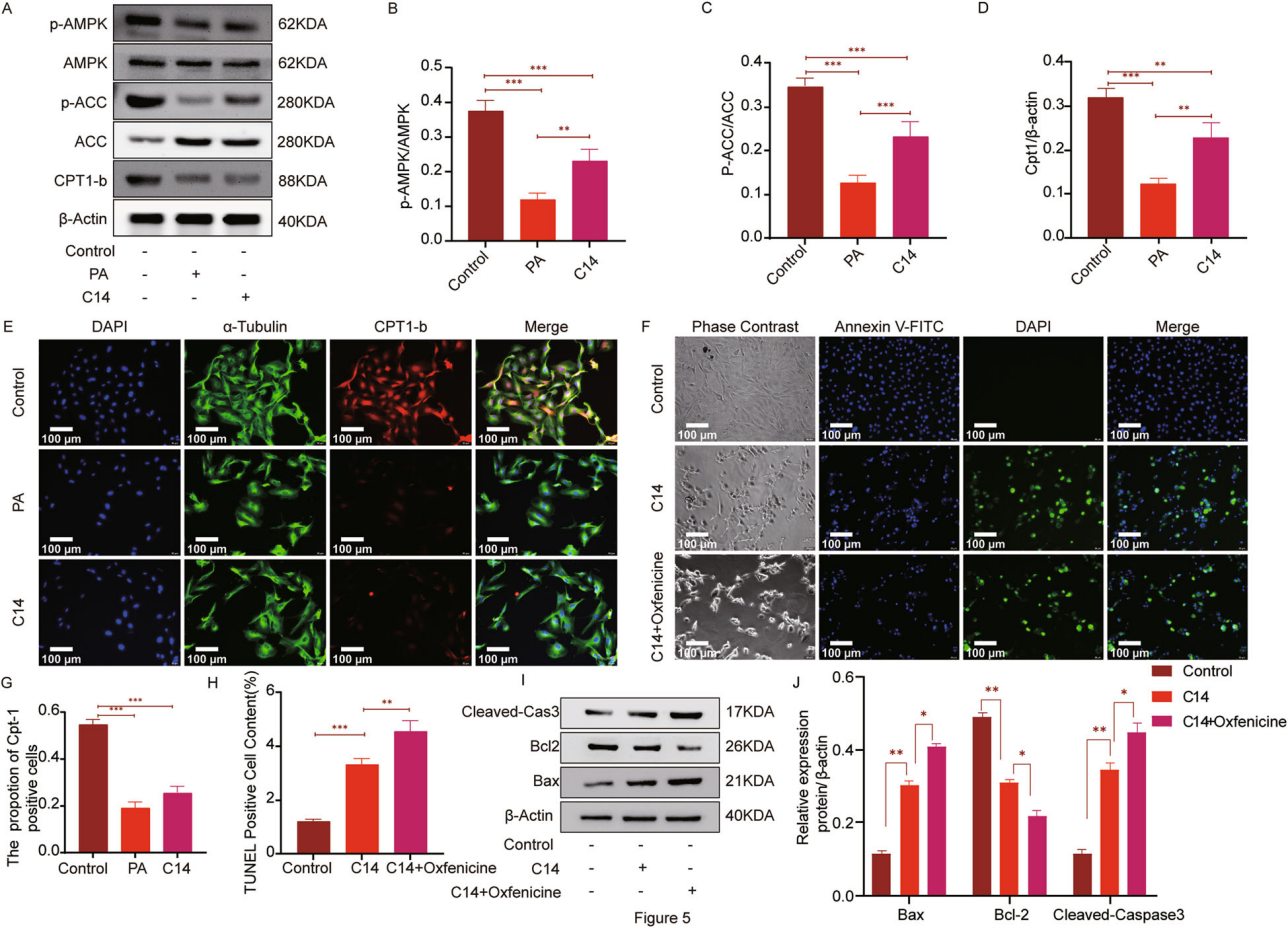
C14 can inhibit the AMPK/ACC/CPT1-b signaling pathway and causes lipid accumulation. **A** The expressions of P-AMPK, AMPK, P-ACC, ACC, and CPT1-b in each group (n = 6), as detected by the western blotting. **B**-**D** Quantification of p-AMPK, AMPK, p-ACC, ACC, and CPT1-b. **E** The levels of CPT1-b in H9c2, as analyzed by Immunofluorescence staining (n = 6, Scale bar = 100 μm). **G** The statistical analysis shows the positive expression of CPT1-b in H9c2. **F**, **H** TUNEL staining and positive cell Quantitative analysis (n = 6, Scale bar = 100 μm). **I**-**J** The expressions of Bcl-2, Bax, and Cleaved-caspase 3 were analyzed by immunoblotting in H9c2 in each group (n = 6). Data are represented as means ± SD, two-way ANOVA followed by Tukey’s post hoc test was used. *P < 0.05, **P < 0.01, and ***P < 0.001

To study the mechanism of C14 affecting H9c2 cells, this research analyzed the expression of fat metabolism-related proteins in H9c2 cells. Compared to the control group, p-AMPK/AMPK, p-ACC/ACC, and CPT1-b in the PA group and C14 group were significantly decreased, as shown by western blotting analysis (Fig. 5A-D). Upon immunofluorescence examination, the expression of cpt1-b in the control group was higher than PA and C14 groups. Additionally, cpt1-b positive cells were also significantly higher in control than PA and C14 groups (Fig. 5E, G). Next, to explore if C14 acylcarnitine requires entering mitochondria to get metabolized, H9c2 cells were intervened with CPT1 inhibitor oxfenicine in-vitro. TUNEL staining analysis showed that C14 + oxfenicine group showed more TUNEL-positive cells in respect to C14 (Fig. 5F, H). Further, western blotting analysis also showed that (Fig. 5I-J), in comparison with C14, the levels of Bax and cleaved caspase 3 in the C14 + oxfenicine group were significantly increased, whereas the expression of Bcl-2 was decreased.

### AICAR reduce lipotoxicity caused by C14

**Fig. 6:**
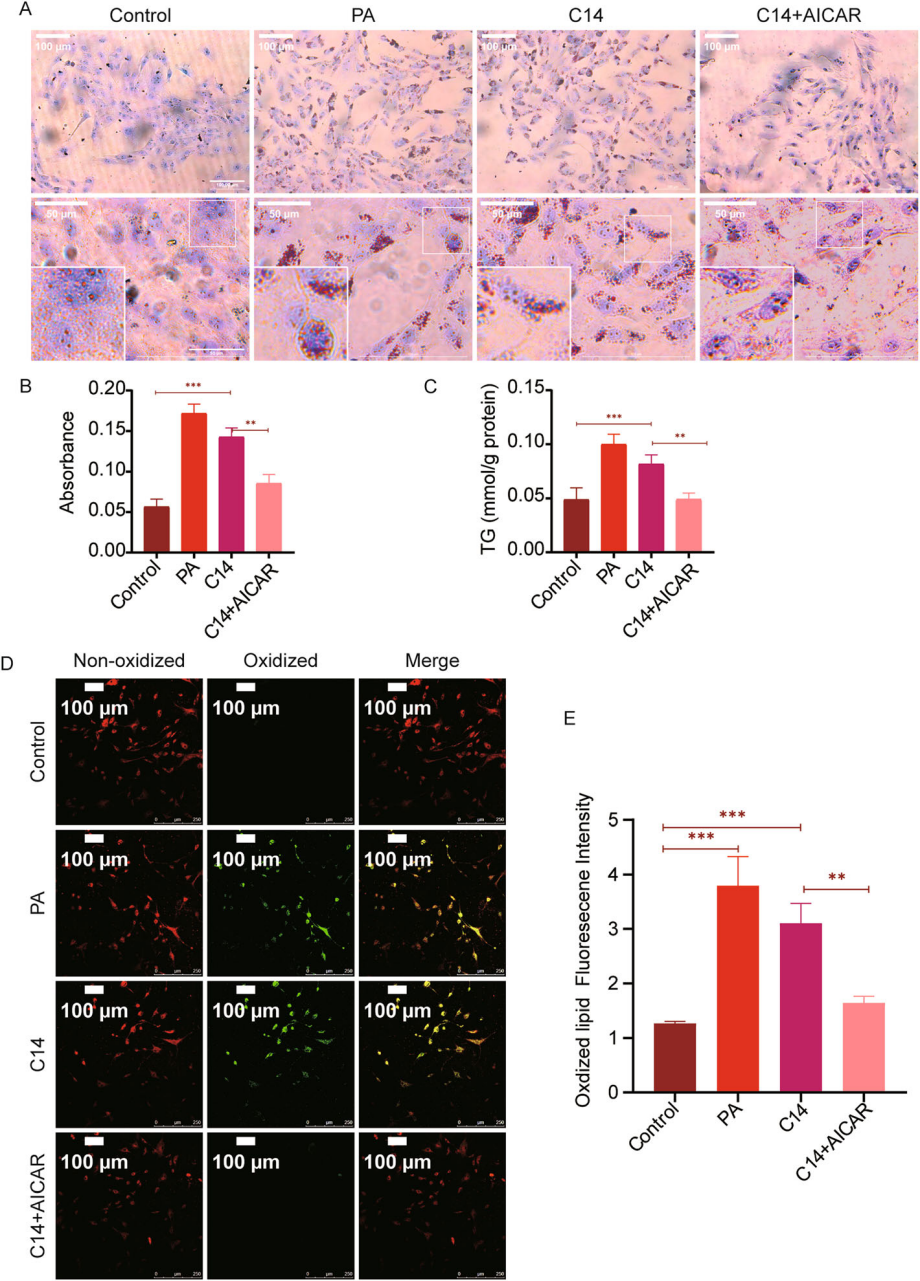
AICAR can reduce lipotoxicity mediated by C14. **A** ORO staining was used to quantify the lipid deposition (scale bar =50 μm, 100 μm). **B** Quantitative analysis of intracellular lipid droplets (n = 6). **C** H9c2 cells in each group were tested for triglyceride (n = 6). **D** Laser scanning confocal microscopy (LSCM) showed the non-oxidized lipid (shown as red) and oxidized lipid (shown as green), H9c2 cells in pretreated with either PA (0.3 mM), C14 (25 μM), or AICAR (1 mM) (Scale bar = 100 μm). **E** Statistical analysis of the fluorescence intensity of oxidized lipid expression from each group (n = 6). Data are represented as means ± SD, two-way ANOVA followed by Tukey’s post hoc test was used. *P < 0.05, **P < 0.01, and ***P < 0.001

The experimental results showed that C14 could inhibit the activity of AMPK (Fig. 5). AICAR, an adenosine analogue, also activates AMPK and thus regulates glucose and lipid metabolism and inhibits the production of proinflammatory cytokines [20]. Therefore, in this study, AICAR was used, as it can penetrate cell membrane AMPK and might neutralize the effect of C14 by reversing the inhibition of AMPK. Oil red staining of cells showed increased lipid deposition in cells in the PA and C14 group increased in varying degrees, whereas the increase in the PA group was higher than the C14 group (Fig. 6A-B). Compared with the C14 group, a reduction in intracellular lipid deposition was observed in the C14 + AICAR group. Statistical analysis showed that intracellular triglyceride content in the PA and C14 groups was significantly enhanced compared to the control group (Fig. 6C). Comparing with the C14 group, intracellular triglyceride content was reduced in the C14 + AICAR group. Additionally, the lipid peroxide content in the control group was lower than the PA and C14 group, while in the C14 + AICAR group, it was lower than the C14 group (Fig. 6D-E).

### AICAR reduces lipid accumulation by activating the C14 inhibited AMPK/ACC/CPT1 signaling pathway

**Fig. 7:**
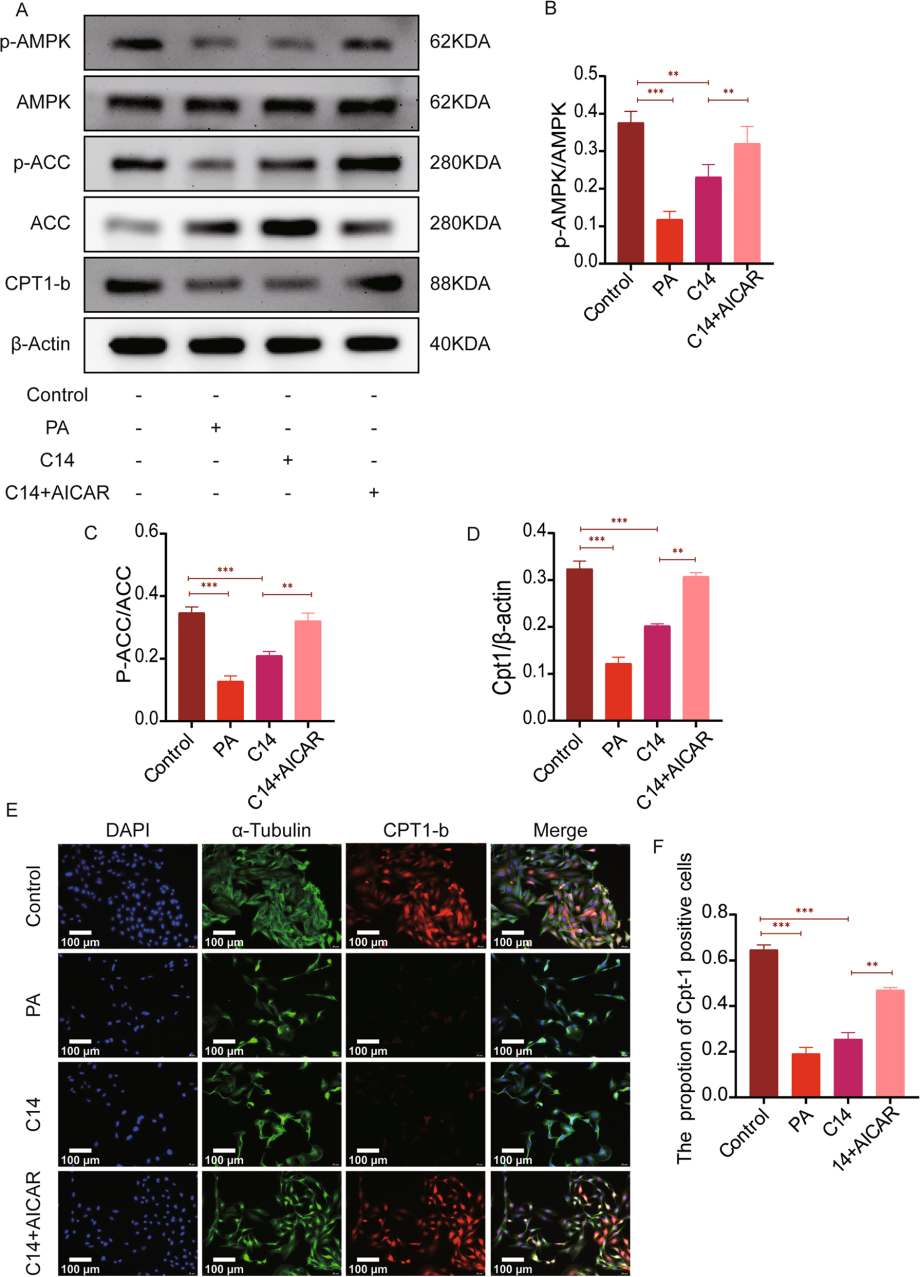
AICAR reduces lipid accumulation by alleviates the C14 mediated inhibition of the AMPK/ACC/CPT1 signaling pathway. **A** Western blotting was used to detect the expressions of P-AMPK, AMPK, P-ACC, ACC, and CPT1-b in each group (n = 6). **B**-**D** Quantification of p-AMPK, AMPK, p-ACC, ACC, and CPT1-b expressions. **E** The levels of CPT1-b in H9c2 were explored by immunofluorescence staining (n = 6, Scale bar = 100 μm). **F** The statistical analysis represents the positive expression of CPT1-b in H9c2. Data are represented as means ± SD, where two-way ANOVA followed by Tukey’s post hoc test was used. *P < 0.05, **P < 0.01, and ***P < 0.001

The levels of p-AMPK/AMPK, p-ACC/ACC, and CPT1-b in the C14 + AICAR group were significantly higher than those in the C14 group (Fig. 7A-D). Also, in the immunofluorescence analysis, the expression of cpt1-b and positive cells in the C14 + AICAR group was significantly increased compared to the C14 group. (Fig. 7E-F).

### AICAR reduces apoptosis caused by C14

**Fig. 8:**
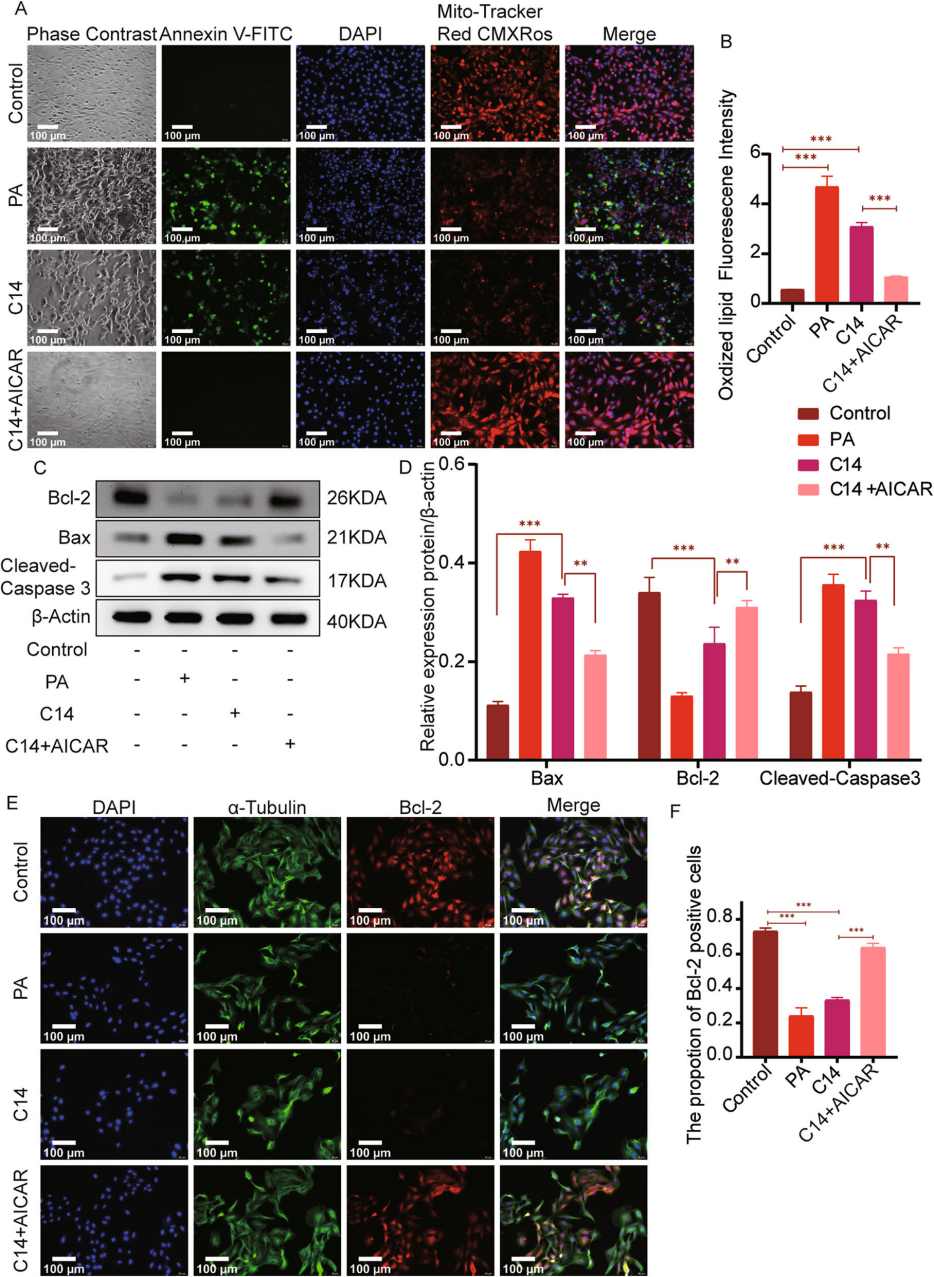
AICAR reduces C14 mediated apoptosis. **A** A fluorescence microscope was used to explore the mitochondrial membrane potential and apoptosis (n = 6, Scale bar = 100 μm). **B** The statistical analysis represents levels of red and green fluorescence (n = 6). **C** The expressions of Bcl-2, Bax, and Cleaved-caspase 3 were analyzed by immunoblotting in H9c2 (n = 6). **D** Bcl-2, Bax, and Cleaved-caspase3 expression results are shown (n = 6). **E** The expressions of Bcl-2 were tested by Immunofluorescence staining (n = 6; Scale bar represents as 100 μm). **F** The statistical analysis represents the positive expression of Bcl-2 in H9c2. Data are represented as means ± SD, two-way ANOVA followed by Tukey’s post hoc test was used. *P < 0.05, **P < 0.01, and ***P < 0.001

Immunofluorescence analysis showed that the PA and C14 group had significant cell apoptosis with decreased mitochondrial membrane potential and morphological changes such as cell, nuclear shrinkage, and nuclear membrane nucleolus fragmentation, compared with the control group. However, upon AICAR treatment to H9c2 cells in the C14 group, these changes were significantly improved, also the apoptosis caused by C14 was alleviated (Fig. 8A-B). The Bax and Cleaved-caspase3 in the PA and C14 groups were substantially higher than the control group, while the expression of Bcl-2 was decreased. Comparing with the C14 group, the levels of Bax and Cleaved-caspase3 in the C14 + AICAR group were significantly reduced, while Bcl-2 levels were greatly improved (Fig. 8C-D). Further, immunofluorescence analysis showed that the expression of Bcl-2 in the C14 + AICAR group was significantly increased than the C14 group, whereas the number of positive cells was also considerably increased than the C14 group (Fig. 8E-F). Altogether, these results indicate that AICAR reduces the apoptosis caused by C14.

### AICAR reduce C14 caused H9c2 cell fibrosis and hypertrophy

**Fig. 9:**
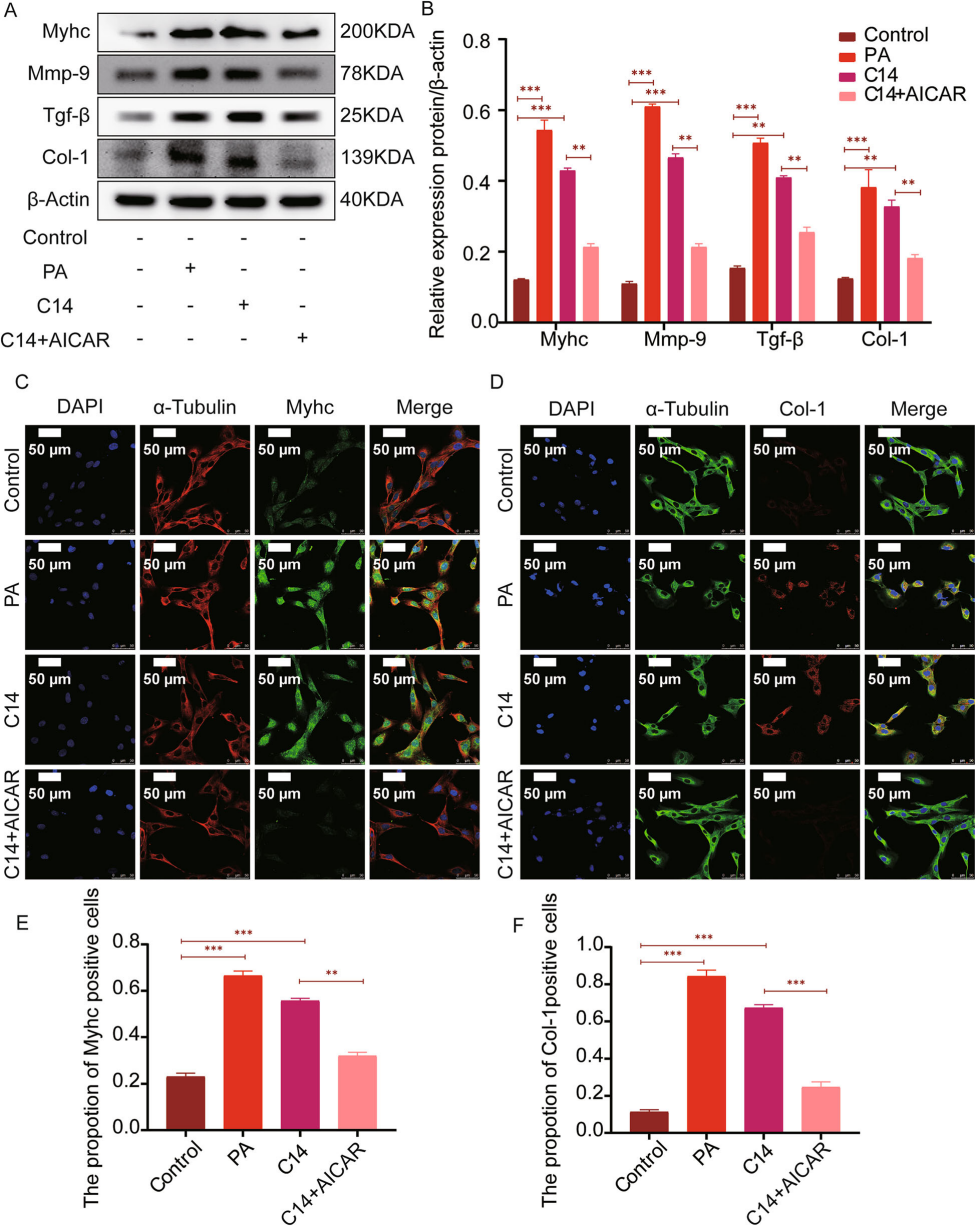
AICAR reduces C14 induced H9c2 cell fibrosis and hypertrophy. **A** The expressions of MYHC, MMP-9, TGF-β, and COL-1 were tested by immunoblotting in H9c2 (n = 6). **B** Quantification of MYHC, MMP-9, TGF-β, and COL-1 expressions (n = 6). **C**-**D** Laser scanning confocal microscopy (LSCM) images show the levels of MYHC and COL-1 in H9c2 (n = 6; Scale bar represents as 50 μm). **E**-**F** The statistical analysis represents the positive expression of MYHC and COL-1 in H9c2. Data are represented as means ± SD, two-way ANOVA followed by Tukey’s post hoc test was used. *P < 0.05, **P < 0.01, and ***P < 0.001

The levels of MYHC, MMP-9, TGF-β, and COL-1 in the PA and C14 groups were significantly higher than those in the control group, as seen through western blotting. Comparing with the C14 group, the levels of MYHC, MMP-9, TGF-β, and COL-1 in the C14 + AICAR group were significantly reduced (Fig. 9A-B). Further, immunofluorescence analysis revealed the expression of MYHC, and the number of positive staining cells in the PA group and C14 group was significantly increased. However, the expression of MYHC and the number of positive staining cells decreased considerably in the C14 + AICAR group compared with the C14 group (Fig. 9C, E). Meanwhile, this research observed that the cell morphology of the PA and C14 group changed, and the expression of COL-1 with the number of positive staining cells was remarkably increased than the control group (Fig. 9D). Compared with the C14 group, COL-1 expression and the number of positively stained cells were significantly decreased in the C14 + AICAR group (Fig. 9F).

## Discussion

DCM is a severe complication associated with diabetes, which adversely affects a patient’s quality of life [21], hence an early diagnosis of DCM is essential. This study investigated the impact of different chain lengths of acylcarnitines with potential association with DCM. By understanding the association of acylcarnitine of varying chain length between ST2DM and DCM, this study intended to predict DCM risk in T2DM patients at an early stage. Therefore, this study analyzed 100 plasma samples (50 cases of DCM and 50 cases of ST2DM) with high throughput metabolomics based on MS targeted metabolomics. This study further explored the potential effect of exogenous acylcarnitine on lipid deposition using the H9c2 rat cardiomyoblast cell model. The analysis showed that factor 1 (C14, C12, C14DC, C14-OH, C20, C18, c16:1-OH) and factor 4 (C8, C6, C10) were positively associated with the risk of DCM (Table 4), which mainly contains the medium- and long-chain acylcarnitines. In cell experiments, the results showed that exogenous C14 supplementation to cardiomyocytes could enhance lipid deposition and further pose obstacles in AMPK signaling pathways, affecting fatty acid oxidation and thus causing myocardial lipotoxicity. These events eventually lead to cardiomyocyte hypertrophy, fibrotic remodeling, and increased apoptosis. Interestingly, these effects were mitigated by the AMPK agonist AICAR. Through this intervention, intracellular lipid accumulation decreased, AMPK signaling pathway, myocardial hypertrophy, and fibrosis were improved, with the reduced cardiomyocyte apoptosis.

Oxidation of fatty acids generally produces acylcarnitine [22, 23], and their accumulation may indicate low β-oxidation and changes in mitochondrial metabolism. However, energy metabolism inactivation flexibility in DCM patients is usually manifested as lipotoxicity. The heart function majorly gets dependent on the oxidation of fatty acids for energy, resulting in excessive fatty acid intake in the body [24, 25]. But mitochondria cannot process many fatty acids, leading to oxidative stress and damage to mitochondrial function [26]. This imbalance of fatty acid uptake with their utilization results in lipid accumulation in the heart [27, 28], and as a result, the heart gets subjected to excessive load, resulting in changes in its structure and function, eventually causing the DCM [29]. Thereby, assessing the dysregulation of fatty acid oxidation in DCM may offer its early prediction. The differential changes of acylcarnitine levels may be used as the potential biomarkers to predict the risk of early impaired glucose tolerance or other diabetic complications. Another study on nonalcoholic liver disease found a positive correlation between the disease state and accumulation of medium and long-chain acylcarnitine [30]. Thus, the medium and long-chain acylcarnitine can be a new screening marker for nonalcoholic fatty liver disease. A study assessing cardiovascular risk in diabetic patients also found that the medium and long-chain acylcarnitines were elevated in T2DM. Many previous reports are in concurrence with the results of the present study, mainly focusing on identifying the differences between medium and long-chain acylcarnitines, for establishing a predictive biomarker marker of diabetes and its complications.

Although many clinical study data strongly support that factor 1 and factor 4 are associated with an increased risk of DCM, however, they did not explore the direct causal relationship between acylcarnitine and changes occurring in myocardial lipid metabolism. Therefore, in the present study, 0.3 mM PA was used to supplement H9c2 cells to establish a model of myocardial insulin resistance to simulate DCM in-vivo. This model does not reflect the long-standing changes associated with diabetes, such as dysglycemia and dyslipidemia. A model of insulin resistance was successfully established as analyzed by measuring the glucose uptake and p-Akt/Akt levels using 2-NBDG (Fig. 3B-C). The tandem mass spectrometry analysis on the control and the PA group showed that the levels of C0, C10, C14, C16, C18, and C22 were significantly increased in C14 (Fig. 3E). In concurrence with the clinical study data, the in-vitro data indicated that the medium and long-chain acylcarnitine were increased in the H9c2 cells with insulin resistance. Next, to further explore the direct causal relationship between the long chain acylcarnitine with the lipid metabolism in cardiomyocytes, the H9c2 cells were stimulated with supplementation of exogenous C14 to examine the role of this acylcarnitine as a potential contributor to ectopic fat accumulation, myocardial remodeling, and increased myocardial apoptosis in cardiac cells. The results showed that C14 induces an increase in lipid storage in H9c2 cells, indicating that C14 might be directly involved in lipid-mediated cardiac dysfunction. To further explore if C14 acylcarnitine needs to enter mitochondria for its metabolism, this research used oxfenicine, a CPT1 inhibitor, to interfere with H9c2 cells. This research found that C14 acylcarnitine was more toxic to H9c2 cells after using oxfenicine, which indicates that C14 acylcarnitine metabolism primarily happens inside the mitochondria (Fig. 5). Next, C14 inhibited the activity of p-AMPK, reducing the activity of p-ACC, inhibiting the oxidation of the fatty acids, and increased the TG and free fatty acids contents in the cells. Finally, increased deposition of lipids in myocardial cells caused lipid metabolism disorders (Fig. 5). AMPK is an essential regulator of cardiac energy homeostasis because its activation induces anti-adipogenesis and promotes fat utilization by regulating the activity of CPT1 [31]. A study reported that CPT-1b deficiency led to cardiac lipotoxicity under pathological stress, causing significant cardiac changes [25, 32]. In this research, the C14 intervention in cardiomyocytes resulted in pro-inflammatory responses, such as an increase in TNFα and IL-1 expression (Fig. 4). Meanwhile, C14 also caused myocardial lipotoxicity, leading to hypertrophic and fibrotic myocardial cells (Fig. 9). Interestingly, the current study demonstrated that, as mentioned earlier, the effect of C14 on cardiomyocytes can be alleviated using AMPK agonist AICAR.

### Comparisons with other studies and addition to the existing knowledge on DCM

Comparing with the previous reports, this study studied the human cohort with and without diabetic cardiomyopathy by analyzing the difference in circulating acylcarnitine by metabolomics. This study found that C14 acylcarnitine was one of the most diverse acylcarnitine species in the diabetic cardiomyopathy cohort. Further, the mechanism of DCM was studied using an in-vitro model, where H9c2 ventricular myoblasts were treated C14 acylcarnitine. Altogether, this research work provides new insights and mechanisms related to the development of ST2DM to DCM.

### Study strength and limitations

The advantages of this research included the transformation method from humans (DCM and ST2DM patients) to experimental models (in-vitro experiments) to eventually translate the findings to the bedside. The specific changes in the medium and long-chain acylcarnitines can provide predictable biomarkers for the disease progression of clinical DCM by helping in its early diagnosis, intervention, and treatment. The clinical method was strictly carried out using the experimental design to avoid any potential interference factors. However, this research also had some shortcomings. This study had a small number of clinical samples, and therefore the study results may have some large deviations, but the results were in concurrence with most previous studies. In the experimental method, this research assessed the effect of acylcarnitine on the myocardium on the cell model. Therefore, further research on the animal model is needed to validate the obtained findings.

## Conclusions

In conclusion, the increased plasma levels of medium and long-chain acylcarnitine extracted from factor 1 (C14, C12, C14DC, C14-OH, C20, C18, C16:1-OH) and factor 4 (C8, C6, C10) were related to the risk of DCM, which indicates that these two factors can be used as the early predictors of DCM risk assessment. The C14 mediated lipid accumulation inhibits the AMPK/ACC/CPT1 signaling pathway, aggravates myocardial lipotoxicity, causes cardiomyocyte hypertrophy and fibrosis, and increases apoptosis. However, these changes were alleviated by the AICAR. From the clinical point of view, this study suggests future treatment strategies for DCM patients for delaying the progression of DCM as well as the early detection of acylcarnitine in patients with T2DM. The real-time monitoring of changes in the medium and long-chain acylcarnitine of DCM can effectively prevent the occurrence of DCM.

## Data Availability

The datasets used and/or analyzed during the current study will be available from the corresponding author on reasonable request.

## Abbreviations

DCM: Diabetic cardiomyopathy
ST2DM: Simple T2DM mellitus
PA: Palmitic Acid
PCA: Principal component analysis
OR: Odds ratio
CI: Confidence interval
NEFA: Nonesterified free fatty acids
AMPK: Adenosine 5′-monophosphate (AMP)-activated protein kinase
AICAR: Acadesine
BMI: Body mass index
FPG: Fasting blood glucose
SBP: Systolic blood pressure
DBP: Diastolic blood pressure
TG: Triglycerides
TC: Total cholesterol
LDL-C: Low-density lipoprotein cholesterol
HDL-C: High-density lipoprotein cholesterol
HbA1c: Glycated hemoglobin
PBG: Postprandial blood glucose
DMEM: Dulbecco’s modification of Eagle’s medium Dulbecco
BSA: Bovine serum albumin
PBS: Phosphate-buffered saline
MTT: 3-(4,5-Dimethylthiazol-2-yl)-2,5-diphenyltetrazolium bromide
ORO: Oil Red O
IQR: Interquartile range
KMO: Kaiser-Meyer-Olkin
TNF-α: Tumor necrosis factor
IL-1β: Interleukin-1β
MMP9: Matrix metallopeptidase 9
CPT1b: Carnitine palmitoyltransferase 1b
Bcl-2: B-cell lymphoma-2
ACC: Acetyl-CoA carboxylase
TGFβ: Transforming growth factor
C0: Free carnitine
C2: Acetylcarnitine
C3: Propionylcarnitine
C4: Butyrylcarnitine
C4-OH: Hydroxylbutyrylcarnitine
C4DC: Succinylcarnitine
C5: Isovalerylcarnitine
C5-OH: 3-Hydroxyisovalerylcarnitine
C5:1: Tiglylcarnitine
C6: Hexanoylcarnitine
C8: Octanoylcarnitine
C10: Decanoylcarnitine
C12: Lauroylcarnitine
C14: Myristoylcarnitine
C14-OH: 3-hydroxyl-tetradecanoylcarnitine
C14DC: Tetradecanoyldiacylcarnitine
C14:1: Tetradecenoylcarnitine
C16: Palmitoylcarnitine
C16-OH: 3-hydroxypalmitoylcarnitine
C16:1-OH: 3-hydroxypalmitoleylcarnitine
C18: Octadecanoylcarnitine
C20: Arachidic carnitine
C22: Behenic carnitine
C24: Tetracosanoic carnitine
C26: Hexacosanoic carnitine

## References

[1] Cho NH, Shaw JE, Karuranga S, Huang Y, da Rocha Fernandes JD, Ohlrogge AW, Malanda B (2018). IDF diabetes atlas: global estimates of diabetes prevalence for 2017 and projections for 2045. Diabetes Res Clin Pract.

[2] Ferrini M, Johansson I, Aboyans V (2019). Heart failure and its complications in patients with diabetes: mounting evidence for a growing burden. Eur J Prev Cardiol.

[3] Lee WS, Kim J (2017). Diabetic cardiomyopathy: where we are and where we are going. Korean J Intern Med.

[4] Jia G, Whaley-Connell A, Sowers JR (2018). Diabetic cardiomyopathy: a hyperglycaemia- and insulin-resistance-induced heart disease. Diabetologia.

[5] McCauley SR, Clark SD, Quest BW, Streeter RM, Oxford EM (2020). Review of canine dilated cardiomyopathy in the wake of diet-associated concerns. J Anim Sci.

[6] Paolillo S, Marsico F, Prastaro M, Renga F, Esposito L, De Martino F, Di Napoli P, Esposito I, Ambrosio A, Ianniruberto M (2019). Diabetic cardiomyopathy: definition, diagnosis, and therapeutic implications. Heart Fail Clin.

[7] Kenny HC, Abel ED (2019). Heart failure in type 2 diabetes mellitus. Circ Res.

[8] Yu W, Gao B, Li N, Wang J, Qiu C, Zhang G, Liu M, Zhang R, Li C, Ji G, Zhang Y (1863). Sirt3 deficiency exacerbates diabetic cardiac dysfunction: role of Foxo3A-Parkin-mediated mitophagy. Biochim Biophys Acta Mol Basis Dis.

[9] Ljubkovic M, Gressette M, Bulat C, Cavar M, Bakovic D, Fabijanic D, Grkovic I, Lemaire C, Marinovic J (2019). Disturbed fatty acid oxidation, endoplasmic reticulum stress, and apoptosis in left ventricle of patients with type 2 diabetes. Diabetes.

[10] Ma S, Feng J, Zhang R, Chen J, Han D, Li X, Yang B, Li X, Fan M, Li C, Tian Z, Wang Y, Cao F (2017). SIRT1 activation by resveratrol alleviates cardiac dysfunction via mitochondrial regulation in diabetic cardiomyopathy mice. Oxidative Med Cell Longev.

[11] Sikder K, Shukla SK, Patel N, Singh H, Rafiq K (2018). High fat diet upregulates fatty acid oxidation and Ketogenesis via intervention of PPAR-γ. Cell Physiol Biochem.

[12] Lorenzo-Almorós A, Tuñón J, Orejas M, Cortés M, Egido J, Lorenzo Ó (2017). Diagnostic approaches for diabetic cardiomyopathy. Cardiovasc Diabetol.

[13] Fisher FM, Maratos-Flier E (2016). Understanding the physiology of FGF21. Annu Rev Physiol.

[14] Yashpal S, Liese AD, Boucher BA, Wagenknecht LE, Haffner SM, Johnston LW, et al. Metabolomic profiling of the dietary approaches to stop hypertension (DASH) diet provides novel insights for the nutritional epidemiology of type 2 diabetes mellitus (T2DM). Br J Nutr. 2021:1–29. 10.1017/S0007114521003561. 10.1017/S0007114521003561 PMC10410496 34511138

[15] Wang Q, Sun T, Cao Y, Gao P, Dong J, Fang Y, Fang Z, Sun X, Zhu Z (2016). A dried blood spot mass spectrometry metabolomic approach for rapid breast cancer detection. OncoTargets Ther.

[16] Geraets I, Chanda D, van Tienen F, van den Wijngaard A, Kamps R, Neumann D, Liu Y, Glatz J, Luiken J, Nabben M (1864). Human embryonic stem cell-derived cardiomyocytes as an *in vitro* model to study cardiac insulin resistance. Biochim Biophys Acta Mol Basis Dis.

[17] Ma Y, Wang W, Devarakonda T, Zhou H, Wang XY, Salloum FN, Spiegel S, Fang X (2020). Functional analysis of molecular and pharmacological modulators of mitochondrial fatty acid oxidation. Sci Rep.

[18] Stuck BJ, Lenski M, Böhm M, Laufs U (2008). Metabolic switch and hypertrophy of cardiomyocytes following treatment with angiotensin II are prevented by AMP-activated protein kinase. J Biol Chem.

[19] Ge MH, Tian H, Mao L, Li DY, Lin JQ, Hu HS, Huang SC, Zhang CJ, Mei XF (2021). Zinc attenuates ferroptosis and promotes functional recovery in contusion spinal cord injury by activating Nrf2/GPX4 defense pathway. CNS Neurosci Ther.

[20] Dagher Z, Ruderman N, Tornheim K, Ido Y (2001). Acute regulation of fatty acid oxidation and amp-activated protein kinase in human umbilical vein endothelial cells. Circ Res.

[21] Yang F, Qin Y, Lv J, Wang Y, Che H, Chen X, Jiang Y, Li A, Sun X, Yue E, Ren L, Li Y, Bai Y, Wang L (2018). Silencing long non-coding RNA Kcnq1ot1 alleviates pyroptosis and fibrosis in diabetic cardiomyopathy. Cell Death Dis.

[22] Nicholas DA, Proctor EA, Agrawal M, Belkina AC, Van Nostrand SC, Panneerseelan-Bharath L, Jones AR, Raval F, Ip BC, Zhu M (2019). Fatty Acid Metabolites Combine with Reduced β Oxidation to Activate Th17 Inflammation in Human Type 2 Diabetes. Cell Metab.

[23] Knottnerus S, Bleeker JC, Wüst R, Ferdinandusse S, IJlst L, Wijburg FA, Wanders R, Visser G, Houtkooper RH (2018). Disorders of mitochondrial long-chain fatty acid oxidation and the carnitine shuttle. Rev Endocr Metab Disord.

[24] Yin Z, Zhao Y, He M, Li H, Fan J, Nie X, Yan M, Chen C, Wang DW (2019). MiR-30c/PGC-1β protects against diabetic cardiomyopathy via PPARα. Cardiovasc Diabetol.

[25] Li Q, Lai X, Sun L, Cao J, Ling C, Zhang W, et al. Antiobesity and anti-inflammation effects of Hakka stir-fried tea of different storage years on high-fat diet-induced obese mice model via activating the AMPK/ACC/CPT1 pathway. Food Nutr Res. 2020;64(0). 10.29219/fnr.v64.1681. 10.29219/fnr.v64.1681 PMC7286352 32577118

[26] Poll BG, Xu J, Jun S, Sanchez J, Zaidman NA, He X, Lester L, Berkowitz DE, Paolocci N, Gao WD, Pluznick JL (2021). Acetate, a short-chain fatty acid, acutely lowers heart rate and cardiac contractility along with blood pressure. J Pharmacol Exp Ther.

[27] Mardani I, Tomas Dalen K, Drevinge C, Miljanovic A, Ståhlman M, Klevstig M, Scharin Täng M, Fogelstrand P, Levin M, Ekstrand M, Nair S, Redfors B, Omerovic E, Andersson L, Kimmel AR, Borén J, Levin MC (2019). Plin2-deficiency reduces lipophagy and results in increased lipid accumulation in the heart. Sci Rep.

[28] Marinho PM, Salomon TB, Andrade AS, Behling CS, Putti JS, Benfato MS, Hackenhaar FS (2019). The effect of n-3 long-chain polyunsaturated fatty acids and lipoic acid on the heart in the ovariectomized rat model of menopause. Free Radic Res.

[29] Sun Z, Zhang L, Li L, Shao C, Liu J, Zhou M, Wang Z (2021). Galectin-3 mediates cardiac remodeling caused by impaired glucose and lipid metabolism through inhibiting two pathways of activating Akt. Am J Physiol Heart Circ Physiol.

[30] Chang Y, Gao XQ, Shen N, He J, Fan X, Chen K, Lin XH, Li HM, Tian FS, Li H (2020). A targeted metabolomic profiling of plasma acylcarnitines in nonalcoholic fatty liver disease. Eur Rev Med Pharmacol Sci.

[31] Jung TW, Lee SH, Kim HC, Bang JS, Abd El-Aty AM, Hacımüftüoğlu A, Shin YK, Jeong JH (2018). METRNL attenuates lipid-induced inflammation and insulin resistance via AMPK or PPARδ-dependent pathways in skeletal muscle of mice. Exp Mol Med.

[32] He L, Kim T, Long Q, Liu J, Wang P, Zhou Y, Ding Y, Prasain J, Wood PA, Yang Q (2012). Carnitine palmitoyltransferase-1b deficiency aggravates pressure overload-induced cardiac hypertrophy caused by lipotoxicity. Circulation.

